# Author Correction: Vaccine elicitation of HIV broadly neutralizing antibodies from engineered B cells

**DOI:** 10.1038/s41467-020-20304-y

**Published:** 2020-12-07

**Authors:** Deli Huang, Jenny Tuyet Tran, Alex Olson, Thomas Vollbrecht, Mary Tenuta, Mariia V. Guryleva, Roberta P. Fuller, Torben Schiffner, Justin R. Abadejos, Lauren Couvrette, Tanya R. Blane, Karen Saye, Wenjuan Li, Elise Landais, Alicia Gonzalez-Martin, William Schief, Ben Murrell, Dennis R. Burton, David Nemazee, James E. Voss

**Affiliations:** 1 Department of Immunology and Microbiology, The Scripps Research Institute, La Jolla, CA USA; 2 Department of Medicine, The University of California San Diego, La Jolla, CA USA; 3 Department of Microbiology, Tumor and Cell Biology, Karolinska Institutet, Stockholm, Sweden; 4 Faculty of Bioengineering and Bioinformatics, Moscow Lomonosov State University, Moscow, Russia; 5 Scripps Consortium for HIV/AIDS Vaccine Development (CHAVD), The Scripps Research Institute, La Jolla, CA USA; 6 IAVI Neutralizing Antibody Center (IAVI), The Scripps Research Institute, La Jolla, CA USA; 7 Faculty of Science, University of Ottawa, Ottawa, Canada; 8 Department of Biochemistry, Universidad Autónoma de Madrid (UAM) and Instituto de Investigaciones Biomédicas Alberto Sols (CSIC-UAM), Madrid, Spain; 9 Ragon Institute of Massachusetts General Hospital, Massachusetts Institute of Technology, and Harvard, Cambridge, MA USA

**Keywords:** Gene therapy, Humoral immunity, HIV infections, Vaccines

Correction to: *Nature Communications* 10.1038/s41467-020-19650-8, published online 17 November 2020.

In this Article, there was an error in the spelling of the name of the first author of reference 32. This author should have been cited as Nahmad, A. D. not as Nahmed, A. D.

This error has been corrected in the HTML and pdf versions of the Article.

