# Supplementary material for: Vaccine elicitation of HIV broadly neutralizing antibodies from engineered B cells
Source: Nat Commun. 2020 Nov 17;11:5850. doi: 10.1038/s41467-020-19650-8 (PMC7673113; doi:10.1038/s41467-020-19650-8)
Supplement: Supplementary file 1 — Supplementary Information [file 41467_2020_19650_MOESM1_ESM.pdf]

Vaccine Elicitation of HIV Broadly Neutralizing Antibodies from Engineered B cells

Huang, D. *et al.*

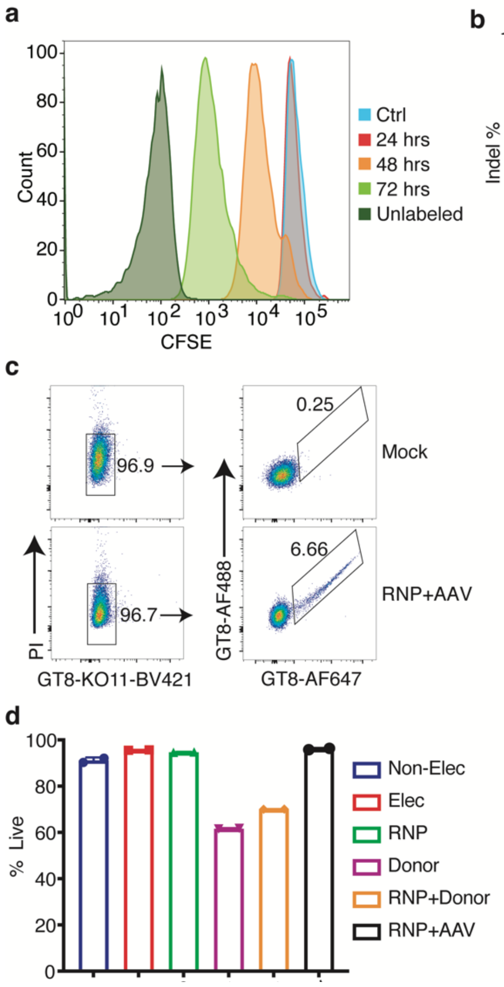

Supplementary Figure 1. Primary B cell engineering

a, **LPS activation.** Because homology directed repair (HDR)-based genome editing requires target cells to be in cycle, the effect of LPS on cell division was assessed to find an appropriate engineering time point. B cells were isolated, labeled with Carboxyfluorescein succinimidyl ester (CFSE), stimulated *in vitro* with LPS (50 μg/ml) and dilution of the CFSE stain intensity that occurs during rounds of cell division was monitored by flow cytometry. Cells had clearly undergone division by 48 h in culture relative to an unactivated control culture. **b**, **CRISPR-cas9 RNP cutting efficiencies.** After 24 hours of LPS culture, *IgH-J4* and *IgK-J5* RNPs were nucleofected individually into cells and gDNA purified 48 hours later. PCR amplicons were generated over the cut sites and sanger sequenced for INDEL quantification by TIDE analysis. n=3 samples in one experiment, **c**, **VRC01 knock-in using *H-targeting* AAV donor DNA**. 24 hours after LPS activation, donor DNA as AAV6 was introduced into cells along with RNPs. Engineering efficiencies were detected using FACS by gating single PI^-,$K011^-$,$eOD\text{-}GT8\text{-}AF647^+$ and $eOD\text{-}GT8\text{-}AF488^+$ cells. The use of two different fluorophore labelled eOD-GT8 proteins eliminated B cells specific for the fluorophores themselves. KO11 was used to gate out non-VRC01 B cells that bind to eOD-GT8. This gating strategy was also used for *H and H+K targeting* using donor DNA plasmid in Fig. 1c. **d, Viability of treated cells after 72 hours.** Viability of electroporated and non-electroporated controls were compared with samples electroporated in the presence of RNP with or without plasmid donor/transduced AAV donors. While engineering efficiencies are similar when *H-targeting* donor DNA is given as plasmid or AAV, AAV delivered donor DNA is significantly less toxic to cells. n=2 samples in one experiment. Data are presented as mean value ± SD in figure b and d.

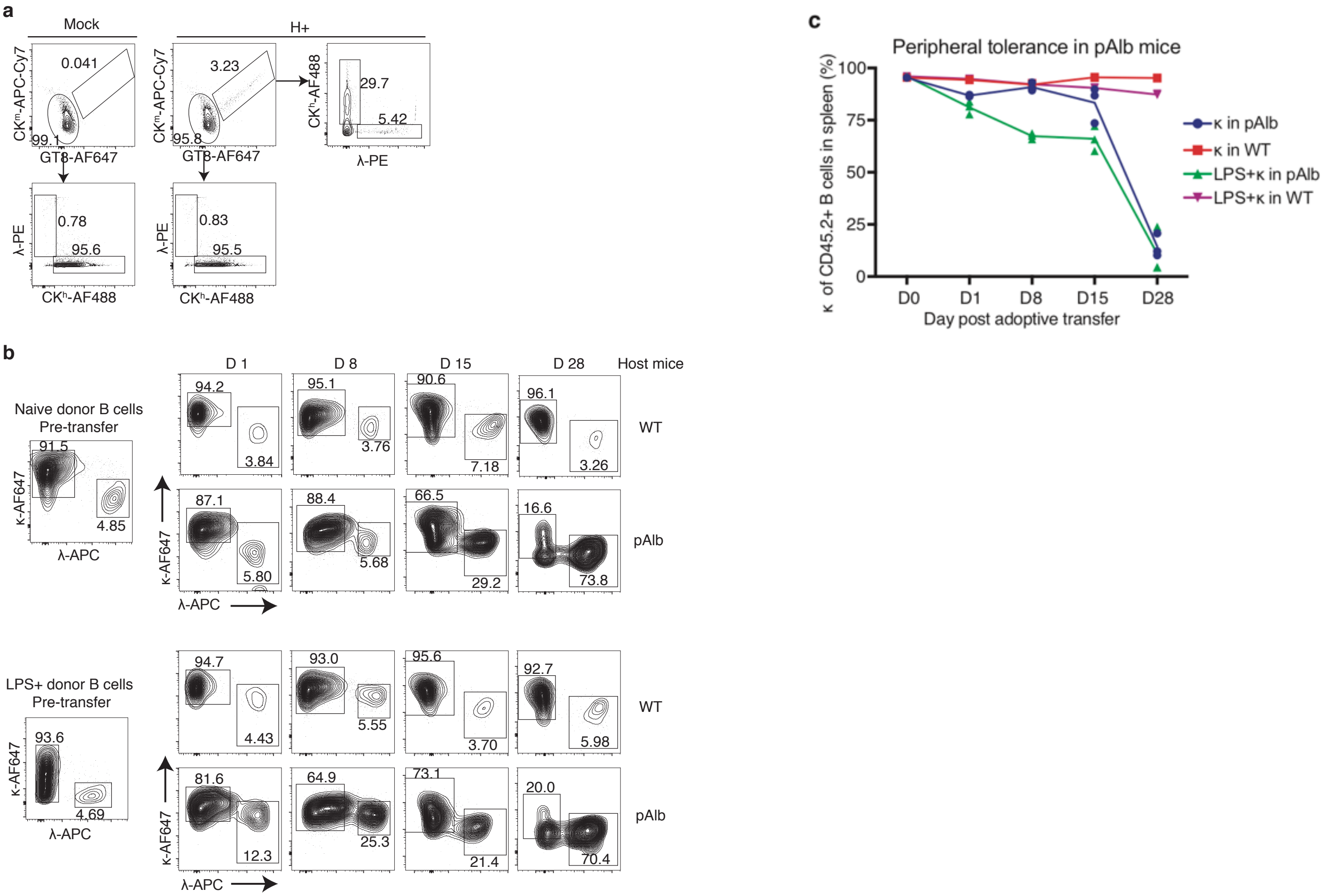

Supplementary Figure 2. Allelic inclusion of *H-targeted* cells and peripheral tolerance *in vivo.* *a, Cell surface expression of endogenous light chains in H-targeted B cells.* *H-targeting* (H$^+$) results in mouse B cells which produce two light chains, the VRC01 engineered mouse kappa chain, and endogenous kappa (95% of cells) or lambda (5% of cells) chains. Pairing of endogenous and engineered antibody chains can result in cell surface expression of auto/polyreactive mixed chain BCRs. To assess surface expression of endogenous light chains in engineered VRC01$^+$ cells, B cells from knock-in mice expressing kappa chains which only use human constant genes were engineered and assessed by FACS. Endogenous (human kappa$^+$/mouse lambda$^+$) light chains were indeed observed on the surface of a significant fraction of VRC01 engineered (GT8$^+$, mouse kappa$^+$) cells, indicating the presence of mixed chain antigen receptors that could be auto/polyreactive. Despite the fact that all successfully *H-targeted* cells should express two light chains, not all VRC01$^+$ cells presented endogenous light chains on their cell surfaces (63% did not). It could be that a significant fraction of the VRC01$^+$ cells express endogenous light chains which are unable to compete with the VRC01 light chain for binding to the VRC01 heavy chain. **b, Auto-reactive B cells are deleted *in vivo* after LPS culture and adoptive transfer.** Untouched or LPS-activated B cells from wild type (WT) animals were transferred to WT or pAlb mice expressing anti-kappa super-antigen in the liver. The fraction of untouched or LPS cultured donor (CD45.2) B cells which were κ^+ after the indicated days post transfer (D1-28) were analyzed by FACS. **c, A summary of results** representing the average values from n=3 animals show that κ^+ cells are depleted from the repertoire when they are autoreactive (in pALB mice) after direct transfer or *ex vivo* LPS activation. Untouched or LPS activated cells were not depleted in κ^+ cells when they were adoptively transferred as non-autoreactive cells into WT mice. This shows that *ex vivo* culture methods required for B cell targeting does not alter subsequent *in vivo* deletion of auto/polyreactive BCRs by peripheral tolerance mechanisms.

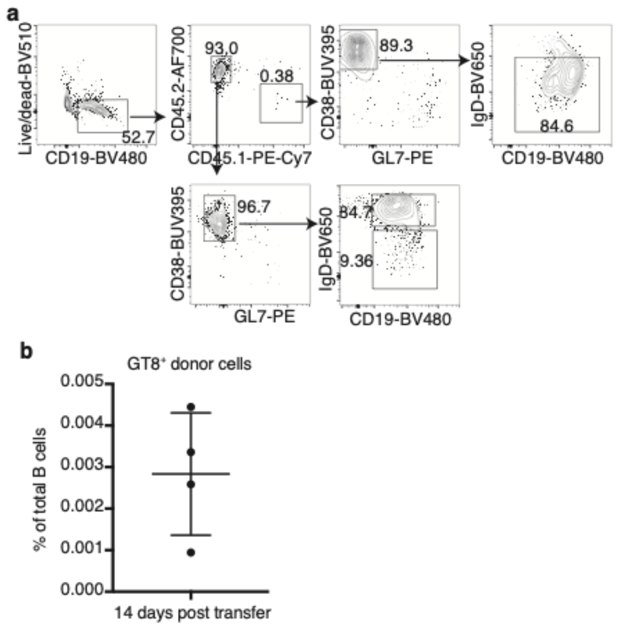

Supplementary Figure 3. Flow cytometry analysis after adoptive transfer of targeted cells.
a, A representative gating strategy for donor memory and naïve B cells. Naïve or LPS-activated engineered B cells (CD45.1) were transferred to WT immunocompetent mice (CD45.2). Host splenocytes are analyzed by flow cytometry 14d post transfer to characterize the phenotype of donor B cells. Splenocytes from mice receiving LPS-activated engineered B cells were chosen to represent memory B cells gating strategy. Memory B cells were gated as CD19$^+$CD38$^+$GL7$^-$IgD$^-$. Naïve B cells are gated as CD19$^+$CD38$^+$GL7$^-$IgD$^+$. Donor and host memory B cells were gated as CD45.1$^+$ and CD45.2$^+$, respectively. Engineered antigen-specific B cells were gated as CD45.1$^+$GT8^{++}KO11$^-$ memory B cells. **b*, Engineered cell frequency *in vivo*.*** Statistical analysis of the frequency of engineered VRC01 cells among splenocyte B cells 14d post transfer. Data are presented as mean value ± SD.

a

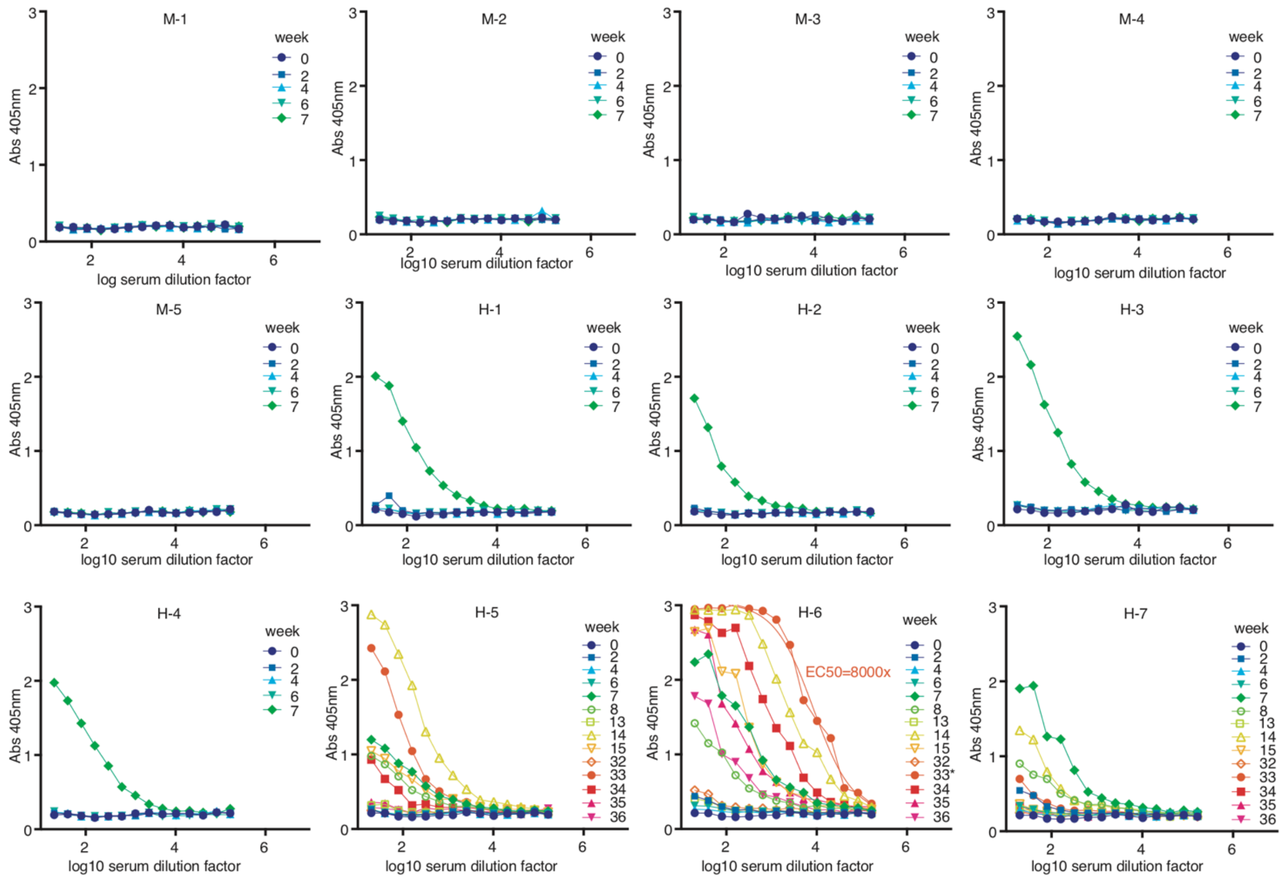

b

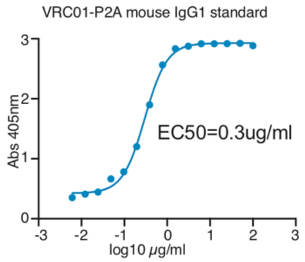

Supplementary Figure 4. P2A ELISA to assess engineered antibody titers in the serum.
Antigen specific IgG in dilutions of mouse serum (x-axis) from the indicated animals (plot title, M=mock, H=*H-targeted*) at the indicated timepoints (plot legends) were captured on MD39-ferritin plates and the P2A peptide on engineered VRC01 LCs was detected using a biotinylated anti-P2A monoclonal antibody and streptavidin-AP. A standard curve was made using a recombinant monoclonal VRC01 LC-P2A tagged mouse IgG1 expressed in 293F cells. For high titer samples with Abs405nm max values =3mAu (the same as the standard), EC50s (in serum dilution factor) were multiplied by the EC50 of the standard (in μg/ml) to quantify the amount of engineered IgG containing the P2A tagged VRC01 light chain in the serum sample.

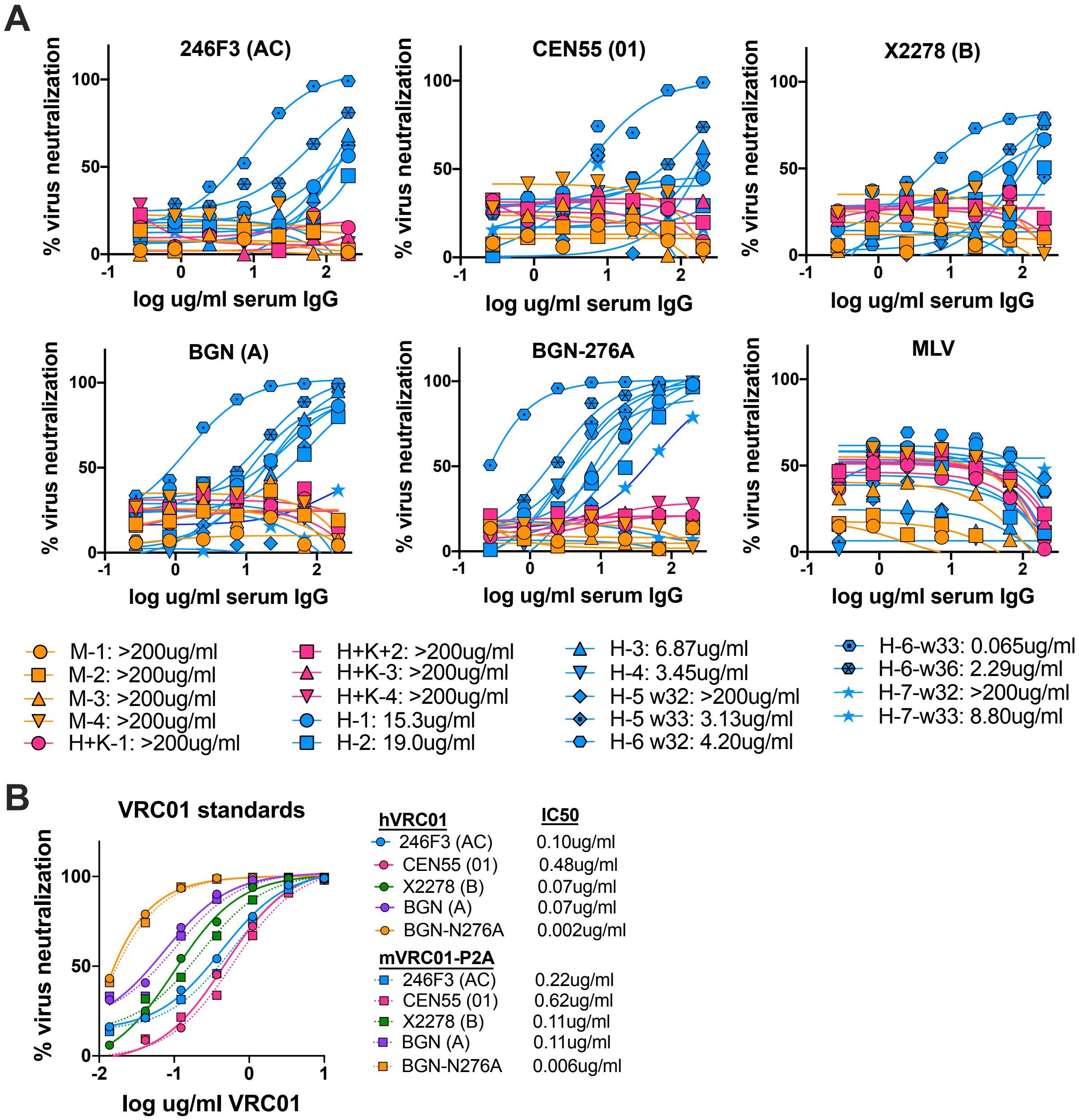

Supplementary Figure 5. Mouse immune serum IgG virus neutralization
a, Serum IgG neutralization. IgG was purified from the serum of the selected samples (Fig. 2f) using protein A/G, and dilutions starting at 200 μg/ml were used in pseudovirus neutralization assays using TZM-bl reporter cells. When neutralization clearly increased with IgG concentration (curves could be fit with positive slopes), % neutralization at 200 μg/ml IgG was reported in figure 2f as a heat map for values between 20 and 100% neutralization. **b, Neutralization of the indicated viruses by the LC-P2A tagged VRC01 mouse IgG1 (mVRC01) or human VRC01 IgG1 (hVRC01) recombinant protein standards** were found to be similar in terms of neutralization IC50s (in μgs/ml) for the indicated viruses. The human mAb is produced by co-transfecting VRC01 kappa and heavy chain plasmids into 293F cells. The mouse VRC01 IgG1 is produced as a single IgG1 transcript where light and heavy chains are cleaved via the intervening P2A peptide as would occur in engineered B cells. The percentage of serum IgG with VRC01 activity for the indicated samples in Fig. 2g was quantified using the following equation: [BGN-N276A IC50 for the mouse VRC01-P2A IgG1 standard (0.006μg/ml)]/[sample BGN-N276A IC50s (given in the legend of a.)]*100.

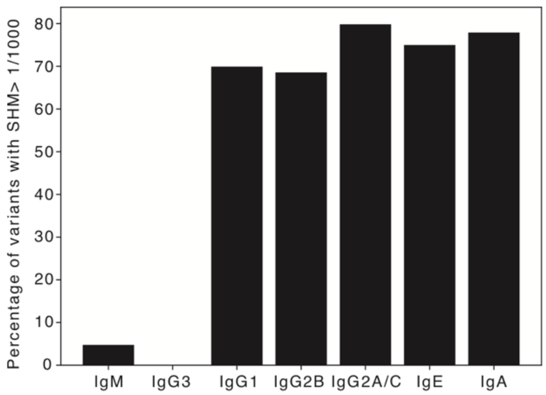

Supplementary Figure 6. Isotype specific mutation in the post-vaccinated repertoire indicates mutations arise from somatic hypermutation. The fraction of sequences from the post-vaccinated repertoires from all animals sequenced which were greater than 0.01% mutated is shown by isotype. IgM and IgG3 sequences showed almost no nucleotide changes compared to donor DNA (<5% for Ig M), while more than 65% of IgG1, G2B, G2A/C, E, and A sequences had >0.01% mutations relative to the donor DNA. These difference were highly significant in a Mann-Whitney U test; IgG3 p-value against IgM=0.39, IgG1 p-value against Ig-M=3.56e-9, IgG2B p-value against IgM=1.35e-8, IgG2A/C p-value against IgM=2.15e-10, IgE p-value against IgM=1.04e-5, IgA p-value against IgM=2.87e-9.

7a

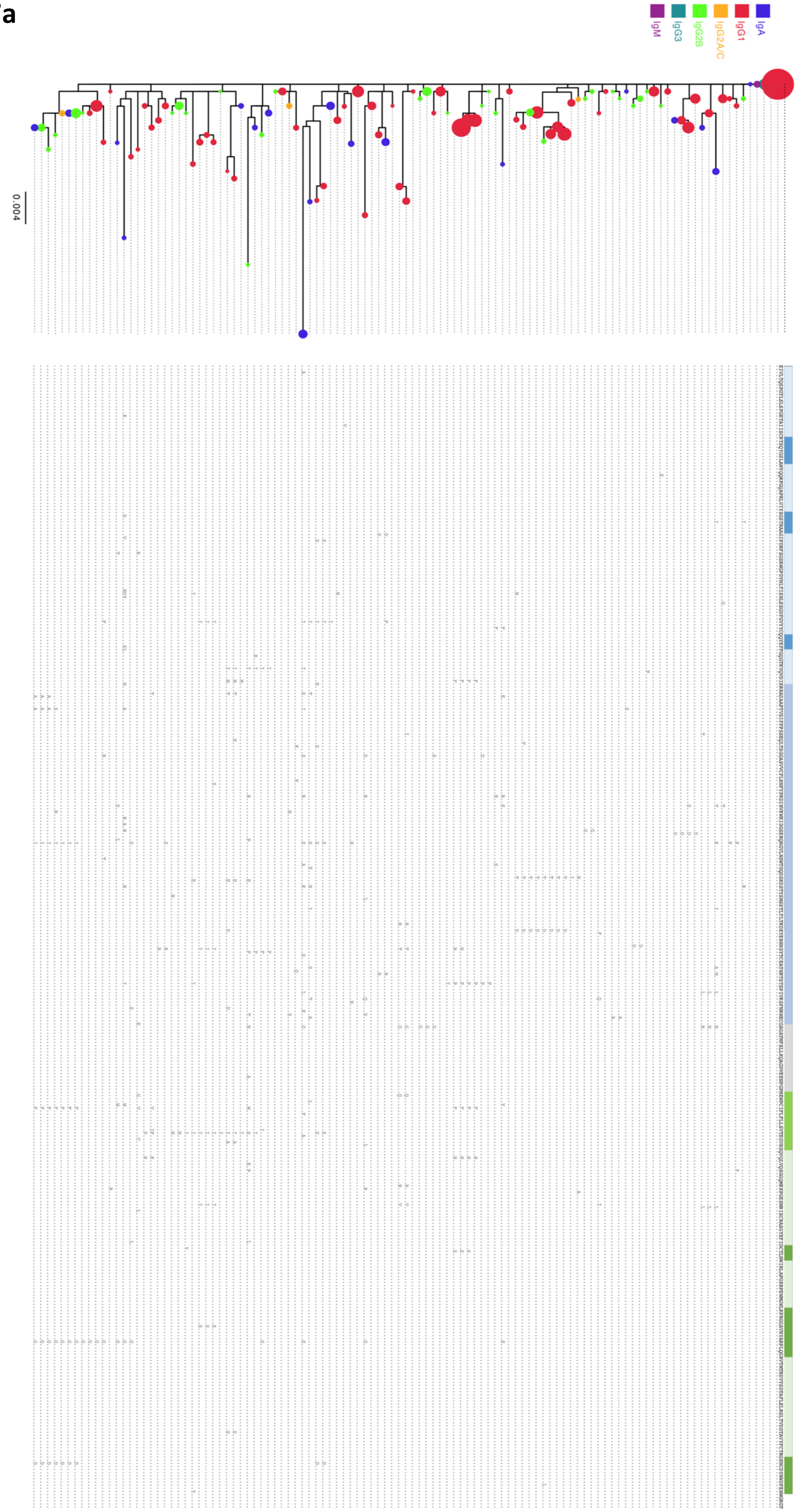

7b

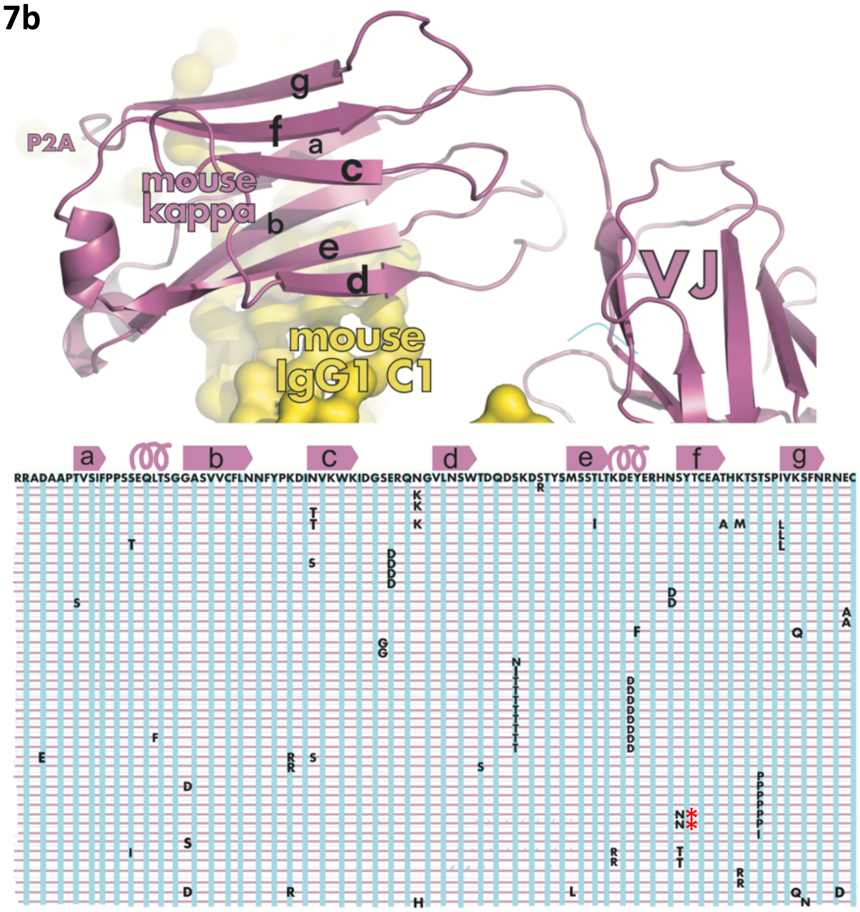

Supplementary Figure 7. Coding changes in the VRC01 gene from a boosted animal
a, All coding sequence changes observed in the engineered repertoire after boosting (from the bone marrow and spleen) are shown along the length of the VRC01 gene corresponding to the indicated clonotype for a representative animal (H-6). **b,** Clonotypes with coding changes within the kappa constant gene are presented with reference to the mouse kappa constant structure (RCSB:1IGY). Most coding mutations accumulate in solvent exposed loops that connect β-strands of the Ig-fold β-barrel. Two of the clonotypes add predicted N-linked glycans indicated with red asterisks.

Vaccination strategy	*ex vivo* activation	Antibody	transferred Ag+ cells	Immunogen	Adjuvant	Mouse No.	TOTAL ANTIGEN SPECIFIC TITERS (EC50)		P2A TITERS (AUC)	
							D14	D56	D14	D56
Resting for 14 days before priming	LPS	VRC01	40,000	MD39-Ferritin	ISCOMIT	1	31.12	3130	0.07413	0.6673
						2	<10	2415	0.0387	0.2154
						3	25.43	174.6	0.04184	0.1386
						4	41.52	36902	0.08437	1.106
						5	39.27	4474	0.03666	0.8968
						6	32.9	7512	0.05557	1.114
						7	35.08	3161	0.04039	0.1287
						8	61.37	35.53	0.04742	0.0597
	CpG+IL4+ Anti CD40					9	44.72	9031	0.05921	2.32
						10	14.04	2614	0.07262	0.5849
						11	<10	1676	0.116	0.5376
			20,000			12	<10	997	0.0607	0.7181
						13	18.24	1922	0.07441	0.4878
						14	37.68	5858	0.1483	0.3105
			4,000			15	3.726	6391	0.05227	1.433
						16	11.22	14871	0.03457	0.5951
						17	42.81	44.62	0.05387	0.07547
	LPS	Mock	40,000		Ribi	18	14.34	5505	0.1288	0.2769
						19	19.21	1319	0.0596	0.1018
						20	16.16	820.5	0.09104	0.139
						21	14.52	233.1	0.1038	0.1528
						22	28.64	6785	0.1124	0.08742
						23	18.78	1102	0.1007	0.06863
						24	18.25	1391	0.05414	0.05151
						25	<10	797.7	0.07221	0.05869
		VRC01				26	46.02	4584	0.05303	0.2591
						27	148.5	5427	0.07147	0.2986
						28	160	5146	0.0669	0.4409
						30	239.9	5699	0.1039	0.4699
						31	362.5	13860	0.07695	0.0719
						32	135.8	3817	0.07722	2.098
						33	75.07	6375	0.04512	0.9299
						34	37.33	1866	0.09172	0.7712
		PGT121				35	31.72	2378	0.04963	0.05431
						36	169.5	12834	0.06643	0.05647
						37	73.33	115.9	0.04402	0.05819
						38	142.2	1976	0.09949	0.09416
						39	1886	31112	0.2077	0.2145
						40	767.5	2693	0.1093	0.08187
						41	603.2	10895	0.0611	0.3749
						42	364.8	10644	0.05952	0.09988
		PGDM1400				43	34.91	561.3	0.09259	0.07807
						44	216.5	1067	0.05135	0.09301
						45	361.1	1767	0.07563	0.06868
						46	124.8	303.5	0.1093	0.08864
						47	24.53	113.9	0.14	0.0835
						48	37.08	4197	0.05912	0.0644
						49	32.13	851.9	0.1382	0.1246
						50	217.7	1153	0.1182	0.08843
		VRC01		eOD-GT8 60mer		51	5654	384644	0.2597	0.04704
						52	1215	112581	0.3898	0.04227
						53	3842	344410	0.1403	0.07415
T-Cell pre-priming		VRC01	65,000	MD39-Ferritin		54	57.92	47987	0.2759	3.619
						55	46	420.1	0.2264	0.4916
						56	216	58912	0.5851	2.87
						57	68.39	9148	0.4625	2.174
						58	38.06	122.3	0.2319	0.06667
						59	90.68	43972	0.07746	2.8
						60	165.1	15140	0.1921	1.673
						61	55.73	10486	0.1396	3.435
				eOD-GT8 60mer		62	21796	388538	3.843	0.0666
						63	2841	241166	2.356	0.04148
						64	3480	307990	2.094	0.06164
						65	15607	911756	0.7895	0.08669
						66	26.46	46754	0.1127	6.81
						67	3083	349055	2.71	0.2387
						68	2999	140720	2.37	0.2298
						69	19147	7862	3.031	0.09359
						70	11160	461068	3.689	0.8612
		Mock	300K FACS ENRICHED	MD39-Ferritin		71	<100	106.6	0.3292	0.2978
						72	<100	<100	0.3055	0.2878
						73	<100	842.6	0.2949	0.3045
						74	<100	1148	0.3026	0.2831
						75	<100	248.6	0.3228	0.2949
						76	<100	<100	0.2684	0.2365
						77	<100	542.3	0.3178	0.2846
						78	<100	289.9	0.3058	0.2444
						79	<100	72.59	0.3033	0.2967
						80	<100	133	0.301	0.2667
						81	<100	<100	0.2588	0.2534
						82	<100	<100	0.2453	0.2159
		VRC01				83	57.18	941.3	0.3007	0.307
						84	113.7	2434	0.2724	0.2592
						85	308.1	2325	0.2562	0.2537
						86	180.8	2429	0.2333	0.2321
						87	<100	5676	0.303	2.688
						88	122.3	570.6	0.2564	0.2767
						89	230.2	2146	0.2494	0.3751
						90	<100	<100	0.2575	0.2767
						91	126.5	5469	0.2604	3.194
						92	152.5	3143	0.2008	1.929
						93	178	3846	0.2507	2.018
						94	212.4	2990	0.2421	2.517

Supplementary Table 1: Engineered B cell vaccine results in other animals. Reproduction or variation of vaccination experiments are shown. Parameters varied are to the left of the animal number. Total antigen specific or engineered (P2A) Ab titers elicited 2 weeks after prime (D14) and 2 weeks after boosting (D56) are shown on the right.
