# Supplementary material for: Vaccine elicitation of HIV broadly neutralizing antibodies from engineered B cells
Source: Nat Commun. 2020 Nov 17;11:5850. doi: 10.1038/s41467-020-19650-8 (PMC7673113; doi:10.1038/s41467-020-19650-8)
Supplement: Supplementary file 3 — Reporting Summary [file 41467_2020_19650_MOESM3_ESM.pdf]

nature research

Corresponding author(s): David Nemazee, Dennis R Burton, James E Voss

Last updated by author(s): Sep 8, 2020

Reporting Summary

Nature Research wishes to improve the reproducibility of the work that we publish. This form provides structure for consistency and transparency in reporting. For further information on Nature Research policies, see our Editorial Policies and the Editorial Policy Checklist.

Statistics

For all statistical analyses, confirm that the following items are present in the figure legend, table legend, main text, or Methods section.

n/a	Confirmed	
☐	☒	The exact sample size (*n*) for each experimental group/condition, given as a discrete number and unit of measurement
☐	☒	A statement on whether measurements were taken from distinct samples or whether the same sample was measured repeatedly
☐	☒	The statistical test(s) used AND whether they are one- or two-sided<br>*Only common tests should be described solely by name; describe more complex techniques in the Methods section.*
☐	☒	A description of all covariates tested
☐	☒	A description of any assumptions or corrections, such as tests of normality and adjustment for multiple comparisons
☐	☒	A full description of the statistical parameters including central tendency (e.g. means) or other basic estimates (e.g. regression coefficient) AND variation (e.g. standard deviation) or associated estimates of uncertainty (e.g. confidence intervals)
☐	☒	For null hypothesis testing, the test statistic (e.g. *F*, *t*, *r*) with confidence intervals, effect sizes, degrees of freedom and *P* value noted<br>*Give P values as exact values whenever suitable.*
☐	☒	For Bayesian analysis, information on the choice of priors and Markov chain Monte Carlo settings
☐	☒	For hierarchical and complex designs, identification of the appropriate level for tests and full reporting of outcomes
☐	☒	Estimates of effect sizes (e.g. Cohen's *d*, Pearson's *r*), indicating how they were calculated

Our web collection on statistics for biologists contains articles on many of the points above.

Software and code

Policy information about availability of computer code

Data collection	LSR II (BD Biosciences), Cytek (Aurora), BD FACSAria (BD Biosciences), SpectraMax Plus (Molecular Devices), Synergy 2 (Biotek), PacBio (Pacific Biosciences)
Data analysis	FlowJo 10.7, Prism 8, , FastTree2, FigTree, Geneious 11.0.5, NGS analysis code and intermediate data processing steps are available at: https://github.com/MurrellGroup/EngineeredBCRseq/

For manuscripts utilizing custom algorithms or software that are central to the research but not yet described in published literature, software must be made available to editors and reviewers. We strongly encourage code deposition in a community repository (e.g. GitHub). See the Nature Research guidelines for submitting code & software for further information.

Data

Policy information about availability of data

All manuscripts must include a data availability statement. This statement should provide the following information, where applicable:

- Accession codes, unique identifiers, or web links for publicly available datasets
- A list of figures that have associated raw data
- A description of any restrictions on data availability

Annotated donor DNA sequence GenBank accession numbers are; MT789856, MT789857 and MT789858. Raw flow cytometry (Figures 1,3, Supplementary Figures 1-3), serological data (Figure 2, Supplementary Figures 4-5, Supplementary Table 1) and processed PacBio data (Figure 4, Supplementary Figures 6, 7) that support the findings of this study are available from the corresponding author J.E.V. upon reasonable request.

April 2020

Field-specific reporting

Please select the one below that is the best fit for your research. If you are not sure, read the appropriate sections before making your selection.

- [x] Life sciences
- [ ] Behavioural & social sciences
- [ ] Ecological, evolutionary & environmental sciences

For a reference copy of the document with all sections, see nature.com/documents/nr-reporting-summary-flat.pdf

Life sciences study design

All studies must disclose on these points even when the disclosure is negative.

Sample size	Small group sizes of 3 or 4 animals were chosen for FACS or serology analysis based on the observation that statistically significant differences could be observed between treatment and control groups using these group sizes. IACUC approval requires that the minimum possible number of animals be used that yield statistically significant results.
Data exclusions	Animals which did not respond to vaccination as measured by total antigen specific antibody titers were excluded from analysis.
Replication	Experiments were repeated and variations of experiments were performed and are reported in Supplementary Table 1.
Randomization	3 month old female mice were randomly assigned to various treatment groups.
Blinding	Investigators were blinded to the assignment of animals into treatment groups and during the generation and analysis of all serological, FACS and sequencing data.

Reporting for specific materials, systems and methods

We require information from authors about some types of materials, experimental systems and methods used in many studies. Here, indicate whether each material, system or method listed is relevant to your study. If you are not sure if a list item applies to your research, read the appropriate section before selecting a response.

Materials & experimental systems

n/a	Involved in the study	
[ ]	[x]	Antibodies
[ ]	[x]	Eukaryotic cell lines
[x]	[ ]	Palaeontology and archaeology
[ ]	[x]	Animals and other organisms
[x]	[ ]	Human research participants
[x]	[ ]	Clinical data
[x]	[ ]	Dual use research of concern

Methods

n/a	Involved in the study	
[x]	[ ]	ChIP-seq
[ ]	[x]	Flow cytometry
[x]	[ ]	MRI-based neuroimaging

Antibodies

Antibodies used	FACS antibibodies: Fc Blocker (homemade mAb 2.4g2) 5ug/ml, anti-CD45.1 (Biolegend, #110728) 1:100 dilution, anti-CD45.2 (Biolegend, #109806) 1:100 dilution, GL7 (Biolegend, #144608, #144610) 1:100 dilution, TCRb (Biolegend, #109228) 1:100 dilution, F4/80 (Biolegend, #123128) 1:100 dilution, Ter119 (Biolegend, #116228) 1:100 dilution, CD38 (Biolegend, #102718) 1:100 dilution, IgD (Biolegend, #405710) 1:100 dilution, IgM (Biolegend, #406512) 1:100 dilution, IgG1 (Biolegend, #406620) 1:100 dilution, CD138 (Biolegend, #142504) 1:100 dilution, Sca-1 (Ly6A/E) (Biolegend, #122512) 1:100 dilution and CD19 (Biolegend, #152408) 1:100 dilution, CD80 (Biolegend, #104712) 1:100 dilution, CD73 (Biolegend, #127210) 1:100 dilution, PD-L2 (Biolegend, #107216) 1:100 dilution, anti-mouse Kappa (Clone 187.1 AF647) 1:100 dilution, Anti-mouse Lambda (Biolegend, #407306) 1:100 dilution, anti-mouse Kappa (BD, #561353) 1:100 dilution, anti-human Kappa (Biolegend, #316506) 1:100 dilution, anti-mouse Lambda (Biolegend, #407308) 1:100 dilution. ELISA/neutralization assay antibodies: alkaline phosphatase-conjugated goat anti-mouse IgG (H+L) (Jackson Immuno Research Labs, #115-055-146) diluted 1:5000, biotin-labeled anti-2A peptide (3H4) mouse antibody (NovusBio, #NBP2-59627) at 1g/ml, VRC01 monoclonal antibody (hVRC01), mVRC01 (homemade), alkaline phosphatase-conjugated goat anti-human IgG, Fc fragment specific (Jackson Immuno Research Labs, #109-055-098) diluted 1:5000. Cell selection antibodies: biotinylated anti-CD45.2 Ab (Clone 104, Biolegend, #109804) 1:100 dilution
Validation	Antibodies obtained commercially have been validated by the manufacturer for the specific use presented in this study. Homemade antibodies have been validated by our lab using appropriate positive and negative controls.

Eukaryotic cell lines

Policy information about cell lines

Cell line source(s)	293T cells were obtained from the American Type Culture Collection (ATCC, #CRL-3216), TZM-bl cells were obtained from the

	National Institutes of Health (NIH) AIDS Reagent Program (#8129)
Authentication	Cell lines were authenticated by ATCC or the AIDS Reagents Program.
Mycoplasma contamination	The cells were not tested for mycoplasma
Commonly misidentified lines (See ICLAC register)	No commonly misidentified lines were used in this study

Animals and other organisms

Policy information about studies involving animals; ARRIVE guidelines recommended for reporting animal research

Laboratory animals	3 month old, female, wild type or pAlb CD45.1/2 Black 6 (C57BL/6) mice were used in this study
Wild animals	no wild animals were used in the study
Field-collected samples	no field-collected samples were used in the study
Ethics oversight	Institutional Animal Care and Use Committee (IACUC) at Scripps Research (La Jolla, CA, USA)

Note that full information on the approval of the study protocol must also be provided in the manuscript.

Flow Cytometry

Plots

Confirm that:

- [x] The axis labels state the marker and fluorochrome used (e.g. CD4-FITC).
- [x] The axis scales are clearly visible. Include numbers along axes only for bottom left plot of group (a 'group' is an analysis of identical markers).
- [x] All plots are contour plots with outliers or pseudocolor plots.
- [x] A numerical value for number of cells or percentage (with statistics) is provided.

Methodology

Sample preparation	Spleen suspensions were generated by smashing the spleen between frosted glass slides. Bone marrow was released from tissue-free tibia and femurs by a mortar-and pestle. Red blood cells were lysed with ammonium chloride (0.83%) before filtering cells through a 40 um cell strainer to generate single-cell suspensions. Fc Blocker (homemade mAb 2.4g2) was added to single-cell suspensions at 0.5 ug per 106 cells before antibody staining. Bone marrow was obtained by crushing tissue-free tibia and femur bones with a mortar and pedestal and rinsing the released cells through a 0.2 um cell strainer into a 50 ml falcon tube. Cells were pelleted by centrifugation (600 x g for 6 min). Red blood cells (RBCs) were disrupted by resuspending the cell pellets in 10 ml of RBC lysis buffer (155 mM NH4Cl + 12 mM NaHCO3 + 0.1 mM EDTA) for 3 min at RT.
Instrument	LSR II (BD Biosciences), Aurora (Cytek), BD FACSAria (BD Biosciences)
Software	FlowJo 10.7
Cell population abundance	B cells were regularly 50% of spleen cells after RBC lysis. Of spleen derived B cells; Total GCBs were between 0.5-5%, memory was between 5-10% and plasma cells were between 1-3%. Donor GCB cells (CD45.1/2) were as low as 0.5% of total GCBs in non-responsive animals after boost (as in immunization of H+K targeted cells) or as high as 35% of GCBs in responsive animals (as in immunization of H-targeted cells). Donor memory made up between 6-20% of total memory after boosting and donor plasma cells were between 1 and 6% of total plasma cells. In the bone marrow, plasma cells made up only between 0,03-0,05% of total cells. Donor cells were between 1-4% of total plasma cells.
Gating strategy	Single lymphocytes were gated using (FSC-A vs SSC-A), (FSC-H vs. FSC-W) and (SSC-H vs. SSC-W) light scattering. Live VRC01 expressing memory cells (MCs) were gated as PI-GT8+KO11-CD19+CD38highslgD-GL7-; Germinal center (GC) B-cells were gated as PI-CD19+ CD38- GL7+; Plasma cells (PCs) were gated as CD138+ Sca1+TCRb-Ter119-slgD-slgM-GL7-F4/80-; Permeabilized surface-stained PCs were intracellularly stained with GT8, KO11 and IgG1 probes. Antibody stains were all diluted 100x into the cell sample.

- [x] Tick this box to confirm that a figure exemplifying the gating strategy is provided in the Supplementary Information.
